# Charles Bonnet Syndrome associated with unilateral vision loss: A new diagnostic perspective

**DOI:** 10.1111/opo.13481

**Published:** 2025-03-18

**Authors:** Giovanni Forte, Natalie Assaf, Paolo Forte, Jasleen K. Jolly

**Affiliations:** ^1^ Vision and Eye Research Institute Anglia Ruskin University Cambridge UK; ^2^ Department of Clinical‐Surgical, Diagnostic and Pediatric Sciences University of Pavia Pavia Italy; ^3^ Eye Unit, IRCCS Ospedale Policlinico San Martino Genoa Italy; ^4^ Jolly Vision Science Cambridge UK; ^5^ Department of Optometry and Vision Science University of Melbourne Melbourne Victoria Australia

**Keywords:** Charles Bonnet Syndrome, monocular, ocular melanoma, sensory deafferentation, visual hallucinations, visual release hallucinations

## Abstract

**Purpose:**

To increase recognition of Charles Bonnet Syndrome (CBS) beyond its conventional association with ‘significant vision loss’, which is indicated in the current literature as a diagnostic criterion.

**Methods:**

Clinical observation of CBS associated with unilateral visual loss following enucleation due to choroidal melanoma. Comprehensive visual assessments were performed. The cognitive function was assessed with the Montreal Cognitive Assessment (MoCA)‐BLIND. The phenomenology, occurrence and impact of visual hallucinations were evaluated using the University of Miami Parkinson's Disease Hallucinations Questionnaire (UM‐PDHQ). A critical literature review of CBS cases associated with vision loss in one eye only was conducted.

**Results:**

In this case and in an additional nine reported cases in the literature, CBS hallucinations occurred following unilateral vision loss despite preserved visual function in the fellow eye. These hallucinations are phenomenologically consistent with those observed after severe bilateral vision loss, indicating that both conditions can lead to the development of CBS.

**Conclusions:**

CBS should be screened in all patients who have experienced any degree of vision loss.

**Key points**

- Charles Bonnet Syndrome can develop following unilateral vision loss, demonstrating that severe bilateral visual impairment is not an essential criterion for the development of the syndrome.
- External medical interventions such as eye patching following ophthalmic surgery can interact with existing vision impairment to trigger Charles Bonnet Syndrome by reducing visual input.
- Healthcare practitioners should implement routine screening for Charles Bonnet Syndrome in all patients experiencing any type or degree of vision loss.

## INTRODUCTION

Charles Bonnet Syndrome (CBS) is a medical condition characterised by complex visual hallucinations secondary to visual loss.^1^ These hallucinations are distinct from those experienced during mental and behavioural disorders, as they occur exclusively following visual loss and patients retain insight into their hallucinatory nature.^2^ Global estimates suggest that CBS may affect up to 47 million people with visual impairment.^3^ This condition often goes unnoticed in clinical practice due to both the lack of awareness among healthcare practitioners and the patients' reluctance to admit to hallucinatory experiences due to the stigma of being labelled as mentally unstable.^4^ Additionally, patients who are unaware that their eye disease can cause visual hallucinations may experience distress due to fear of having severe mental health conditions, such as psychosis or dementia.^2^ Notably, in one‐third of cases, CBS has a negative impact on patients' lives, and this condition has been defined by Cox and ffytche^5^ as ‘negative‐outcome CBS’.

In the International Classification of Diseases (ICD‐11), CBS is classified as ‘visual release hallucinations’, a term derived from the most accepted pathogenetic theory known as the ‘perceptual release theory’.^6^ This theoretical framework, initially proposed by West and subsequently elaborated by Cogan, established the foundational concept that the loss of visual input triggers spontaneous hyperexcitability in visual areas, resulting in visual hallucinations.^7^ The underlying mechanism can be conceptualised both in cognitive‐psychological terms as a ‘release’ phenomenon and in neurophysiological terms as ‘deafferentation’.^2^, ^7^, ^8^ The precise mechanism by which deafferentation of relevant cortical areas leads to visual hallucinations remains unclear.

The phenomenological framework of CBS was delineated by ffytche.^9^ A distinctive feature of hallucinations in CBS is that they are exclusively visual, occurring without involvement of other sensory modalities. The frequency of visual hallucinations varies considerably among patients. Each episode typically lasts seconds to minutes. Generally, hallucinations are more frequent in the weeks and months after their initial manifestation and tend to be self‐limiting. Approximately three‐quarters of patients continue to experience hallucinations 5 years after diagnosis, although with decreased frequency and duration.^5^ The hallucinations may be stationary or moving, often in colour, and may be localised to specific visual field regions.^10^ Hallucination content can be categorised into simple and complex types. Simple hallucinations include elementary phenomena such as lights, flashes, colours, dots and basic shapes, as well as geometric patterns like grid patterns, brickwork, lattices and tapestries, known in the literature as ‘tessellopsia’.^2^ Complex visual hallucinations encompass human figures and faces, animals, objects and panoramic landscape scenes. Individual patients typically experience only a subset of these manifestations, and the content may vary across episodes. In clinical practice, it is important to document carefully the hallucination content, which presents distinctive characteristics. In CBS, human figures appear silent and do not interact with the patient, typically presenting with unusual features like elaborate headwear. These figures often wear costumes such as military attire or appear draped in cloaks, and may be diminutive in size, with patients reporting them as children or Lilliputian figures.^2^ Additionally, facial features may appear distorted, a condition known as prosopometamorphopsia.^11^

The majority of authors do not apply a specific set of diagnostic criteria, but rather work under the assumption that CBS refers to complex visual hallucinations in visually impaired individuals with insight, without secondary delusional elaboration.^1^ CBS can develop from any condition that impairs visual signal transmission along the visual pathway, making all ocular diseases potential triggers for this syndrome.^2^ The current literature highlights the notion that a ‘significant visual loss’, defined as a loss of vision exceeding 60%, is necessary to develop CBS.^12^, ^13^ As a consequence of this paradigm, most CBS studies and case reports have focused on patients with advanced bilateral ocular pathologies such as macular degeneration, geographic atrophy, diabetic retinopathy, glaucoma and cataract.^14^

The case detailed here presents CBS associated with acute unilateral loss of vision following enucleation due to melanoma, which contradicts this notion, as the fellow eye maintained excellent visual function. A critical review of the literature was conducted to compare whether the phenomenology of visual hallucinations in cases of CBS associated with vision loss in one eye only differs from that in cases where the ‘significant visual loss’ criterion is met (i.e., severe bilateral age‐related macular degeneration, advanced glaucoma).

The aim of this paper is to underscore the importance of broadening the recognition and understanding of CBS beyond its conventional association with severe bilateral visual impairment.

## METHODS

Presentation of a clinical observation of CBS following unilateral visual loss. Phenomenology, occurrence and impact of visual hallucinations were evaluated using the University of Miami Parkinson's Disease Hallucinations Questionnaire (UM‐PDHQ).^15^ A complete ophthalmic examination was performed. Visual acuity testing was completed using the Freiburg Acuity Test (FrACT) (michaelbach.de/fract/).^16^ Additionally, central retinal sensitivity was measured using MAIA microperimetry with a 10–2 grid, without formal dark adaptation or dilation (ICare CenterVue; icare‐world.com/).^17^ Cognitive function was assessed with the Montreal Cognitive Assessment (MoCA)‐BLIND.^18^

In order to assess whether this was an isolated case, a literature review of other reports of CBS associated with loss of vision in one eye only was conducted, where normal visual function in the fellow eye was confirmed. The research of literature was performed up to 28 June 2024 by searching the electronic database PubMed®/MEDLINE. The search was restricted to case reports and case series written in English or Italian. Articles written in other languages with at least an English or Italian abstract were also considered if adequate information could be captured from the abstract. Articles without an English or Italian abstract were excluded.

The Rayyan program (Rayyan Systems Inc.; rayyan.ai/) was used to organise and manage the collaborative literature review.^14^ Two authors (GF and PF) independently examined the title and abstracts of all identified records and removed those deemed as obviously irrelevant. Two authors (GF and NA) then independently examined the remaining references in full text for eligibility. All selected works were critically analysed, and the associated reference sections were carefully screened to identify other pertinent articles. Disagreements in study selection were discussed between the authors, and where consensus could not be reached, a third author (JKJ) was included in the final decision‐making. The cases that met the inclusion criteria were reviewed critically. Table 1 details the literature search keyword terms and the screening inclusion and exclusion criteria.

**TABLE 1:** The literature search terms and screening exclusion and inclusion criteria to conduct the research.

**Search term**
Charles Bonnet
Charles Bonnet Syndrome
Charles Bonnet Syndrome (CBS)
**Screening exclusion criteria**
Bilateral eye diseases/vision loss
Insight is not retained
Primary or secondary delusions
Hallucinations in other modalities
Primary neurological diseases
Significant cognitive impairment
**Screening inclusion criteria**
Diagnosis of CBS secondary to visual loss in one eye only^a^
Confirmation of normal visual function in fellow eye
*Fellow eye visual acuity*
Best corrected visual acuity (BCVA) of 6/6, 20/20 or 0.00 logMAR
Standard visual acuity (VA) better than 6/12, 20/40 Snellen fraction or + 0.30 logMAR^b^

^a^ Without any restriction on the method for the diagnosis of CBS.

^b^ Not specified whether VA was best corrected or uncorrected.

## RESULTS

### Case presentation

In May 2024, the patient volunteered for a research study and consented to further investigation (PSY‐S21‐007). Informed consent was provided, and all assessments adhered to the Declaration of Helsinki.

A 67‐year‐old female had a 2‐year history of CBS. The patient had good cognitive function with a score of 19 on the MoCA‐BLIND and no history of psychiatric illness.^18^ After a diagnosis of choroidal melanoma in her right eye (in 2021), she underwent an uncomplicated enucleation in March 2022. The hallucinations began the day after the operation and have occurred frequently without abatement since then.

**FIGURE 1:**
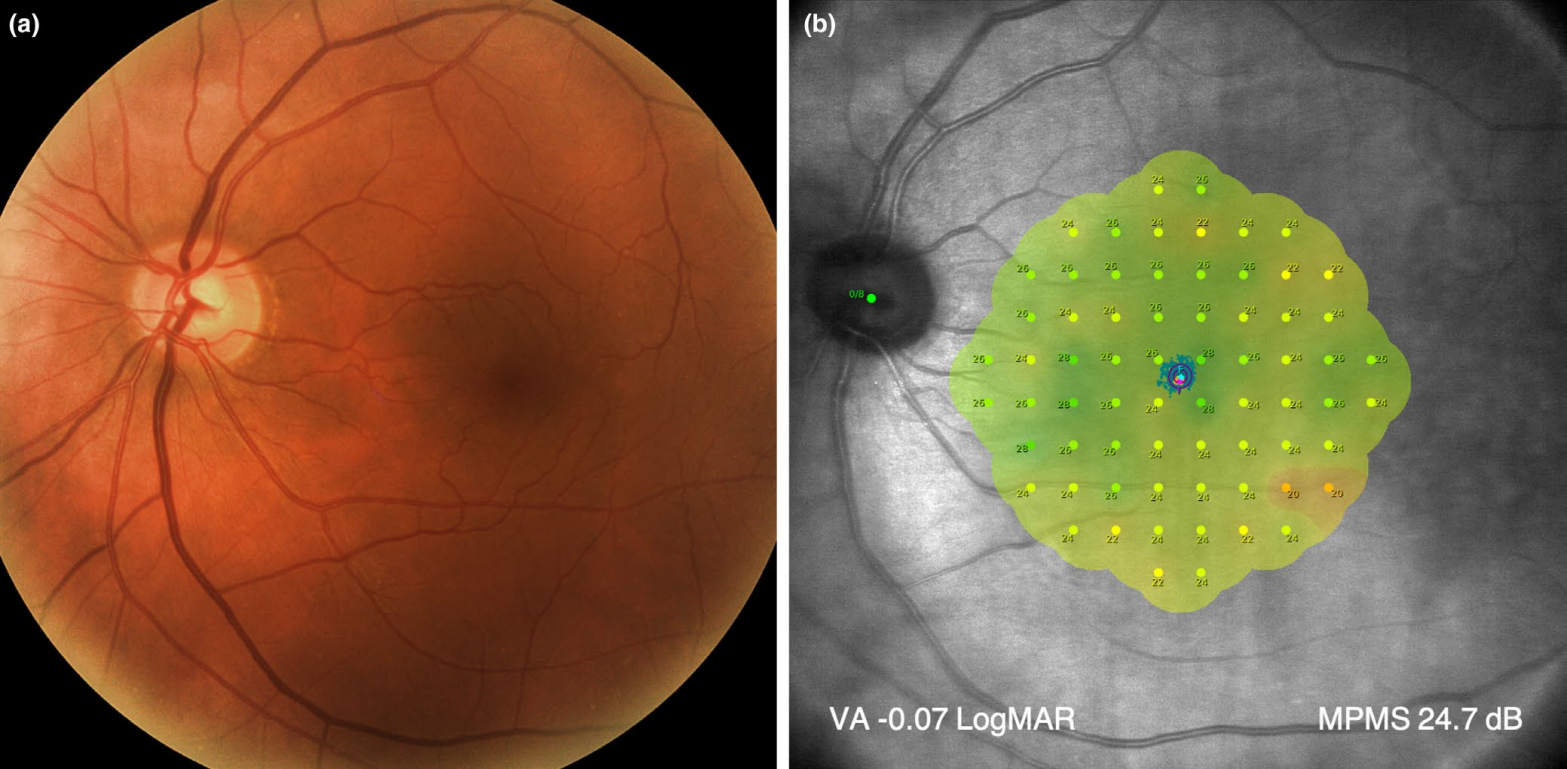
Left eye. (a) Colour fundus photograph. (b) Microperimetry plot results, standard VA with a range of central retinal sensitivity function. The brighter colours on the threshold sensitivity map indicate greater central function. MPMS, microperimetry mean sensitivity; VA, visual acuity.

The fellow eye exhibited visual function within normal limits. Visual acuity was −0.07 LogMAR in the left eye, and microperimetry mean sensitivity was 24.7 dB (Figure 1b). No central or peripheral retinal or optic nerve abnormalities were detected upon fundus examination (Figure 1a).

**FIGURE 2:**
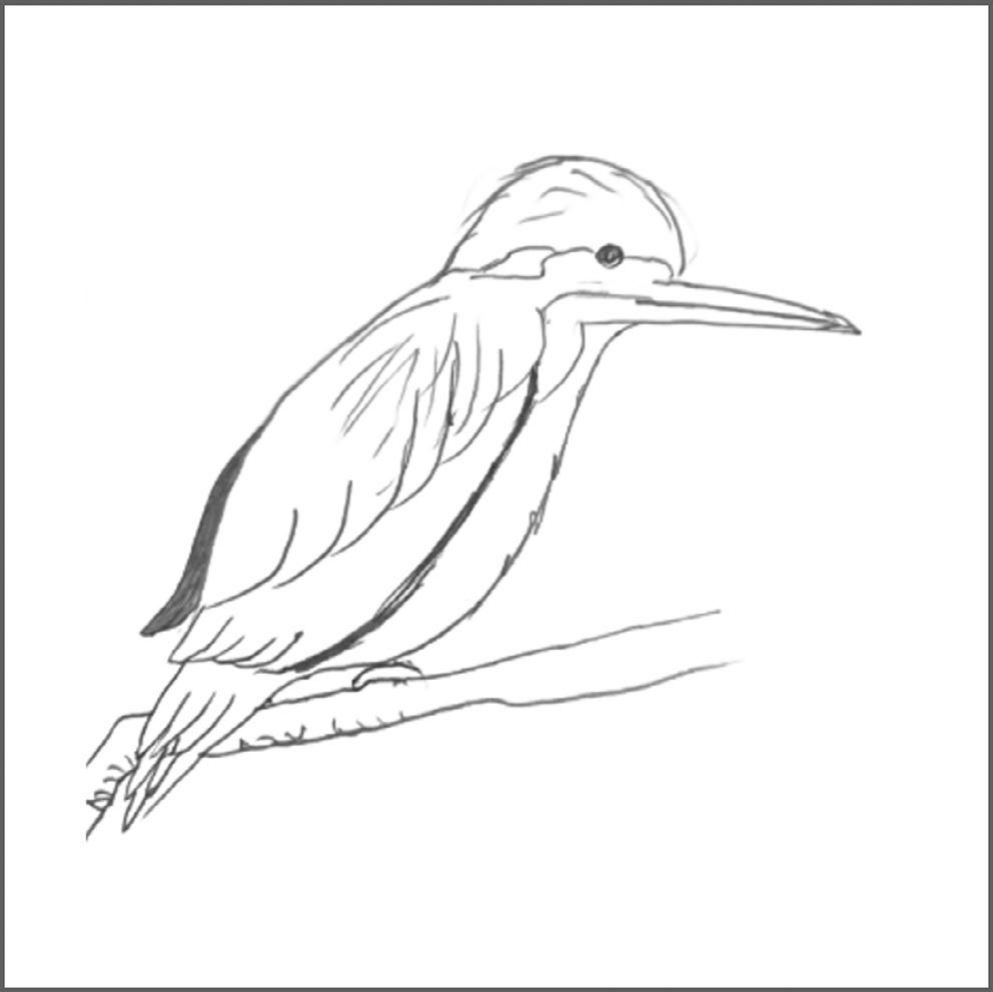
Patient's graphic depiction of kingfisher hallucination.

The patient was prompted to describe the images that she perceived. To facilitate this, the UM‐PDHQ was conducted to provide an overall insight into the hallucinations.^15^ The patient described having very frequent visual hallucinations occurring several times per week, with each hallucination lasting for more than 10 s. The patient experienced several types of visual hallucinations consisting of multiple types of images that were of normal size, coloured and solid. The patient expressed that she was aware that these visual images were not real. The visual images consisted of constantly moving patterns and flashing lights of different colours. She also saw a fire, a fully decorated Christmas tree with lights, a robot that sits between her and her husband in the car, kingfishers and a windmill. She sees images of whole people, such as dark dead bodies, on car bonnets. The most recent images were a lunchbox, household items, mundane objects, a chair in the bedroom and lights/patterns of lights. When asked about how distressing she found these images to be, she mentioned that they were mildly distressing. The patient described the visual hallucinations as having a sudden onset, occurring mostly in dim light. At our request, the patient provided a depiction of the kingfisher hallucination as she perceived it (Figure 2).

### Literature review

**FIGURE 3:**
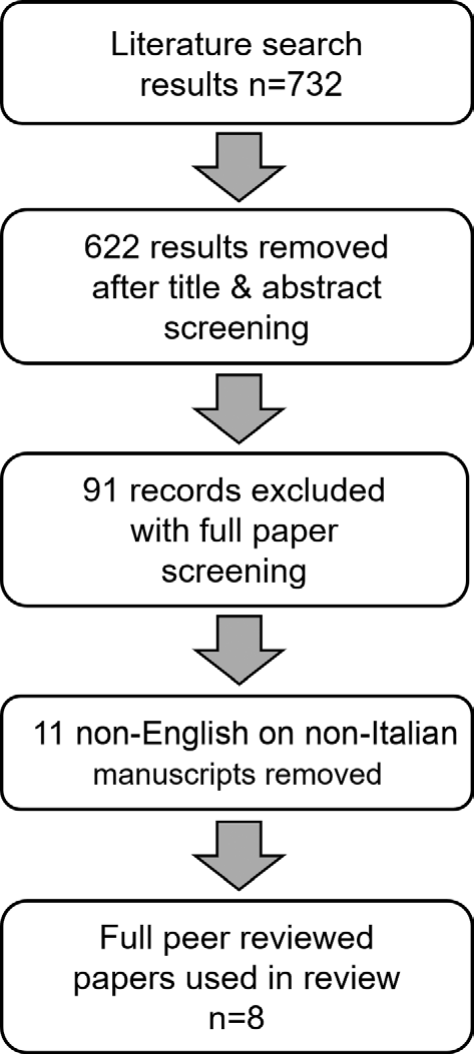
The Preferred Reporting Items for Systematic Reviews and Meta‐Analyses (PRISMA) diagram: Literature search, screening process and results.

The literature search showed 732 results; 724 results were excluded following abstract or full paper screening; consequently, eight peer‐reviewed articles were included in the final analysis (Figure 3). The types of studies were six case reports and two short case series. Of the two case series by Tan et al.^19^ and Beaulieu et al.^20^ 1/2 and 2/3 cases met the inclusion criteria respectively. Thus, in total, the literature search identified nine CBS cases associated with visual loss in just one eye, demonstrating that the presented case is not an isolated clinical observation. Detailed demographic information and a summary of CBS aetiology for each case are provided in Table 2. Fydanaki and Joganathan^21^ did not report the gender of the patient. Other cases included six males and two females, with a male‐to‐female ratio of 3:1. The mean age at diagnosis was 69.4 years (range 52–82).

**TABLE 2:** Main characteristics of the nine cases of Charles Bonnet Syndrome previously described in the literature that met the inclusion criteria for the review, listed in order of publication date (2005–2024).

Authors	Age, gender	Ophthalmic association	Visual sensory deprivation	Eye	Fellow eye VA^a^	VH onset	VH cessation
Ross and Rahman^22^	65, M	Enucleation following choroidal melanoma	None	R	6/6^b^	3 mo after surgery	NA
Tan et al.^19^	63, F	CRAO	None	L	20/30^b^	2 d after onset of symptoms	14 wk. after onset of symptoms
Khadavi et al.^23^	70, M	Cicatricial ectropion	Eye patch following surgery	R	20/20^c^	2 d postoperatively	2 d following patch removal
Gander et al.^24^	68, M	Dislocated orbital floor fracture	Frost suture following surgery	L	+0.3^c^	3 h postoperatively	2 h after removal of the Frost suture
Wilson et al.^25^	69, M	Eyelid basal cell carcinoma and cicatricial ectropion	Reconstruction with a Hughes procedure following surgery	R	20/25^b^	5–7 h postoperatively	4 days postoperatively, despite ongoing monocular occlusion
Beaulieu et al.^20^	78, M	Lower eyelid retraction and ectropion	Eye patch and Frost suture following surgery	L	20/20^b^	Few hours postoperatively	2 d after the removal of the patch and Frost suture
Beaulieu et al.^20^	82, M	Cicatricial ectropion and exposure keratopathy	Eye patch and Frost suture following surgery	R	20/30^b^	2 d postoperatively	Immediately after the removal of the patch and Frost suture
Fydanaki and Joganathan^21^	52, NA	CRAO	None	L	6/6^b^	2 d after vision loss in LE	5 d after vision loss in LE
Woods et al.^26^	78, F	Optic neuritis	None	R	20/25^b^	Following vision loss in RE	NA

Abbreviations: CRAO, central retinal artery occlusion; d, day; F, female; h, hour; L, left; LE, left eye; M, male; mo, month; NA, not available; R, right; RE, right eye; VA, visual acuity; VH, visual hallucinations; wk., week.

^a^ Snellen or logMAR.

^b^ Not specified if VA was best corrected or uncorrected.

^c^ Uncorrected VA.

Due to the lack of adequate clinician training and clear guidelines on specific questions to ask a patient presenting with CBS, not all reports included important information for a comprehensive evaluation. Therefore, this report will focus primarily on the aspects that were uniformly reported in all cases: ophthalmological associations, time course, progression and the phenomenology of hallucinations.

The ophthalmic pathologies associated with CBS are listed in Table 2. The hallucinations emerged following a spectrum of conditions, including enucleation,^22^ visual pathway diseases (unilateral optic neuritis),^26^ retinal diseases (central retinal artery occlusion, CRAO),^19^, ^21^ anterior segment pathologies (exposure keratopathy),^20^ orbital disorder (dislocated orbital floor fracture)^24^ and eyelid disease (basal cell carcinoma, cicatricial ectropion, eyelid retraction).^20^, ^25^

Regarding the onset of hallucinations, in 8/9 cases, they manifested within a minimum of a few hours to a maximum of 2 days following unilateral vision loss. In contrast, Ross and Rahman^22^ reported a case where visual hallucinations began 3 months after an uncomplicated enucleation due to choroidal melanoma. The resolution of hallucinations, documented in 7/9 cases, exhibited a range of timelines. Fydanaki and Joganathan^21^ and Tan et al.^19^ reported visual hallucination resolution 5 days and 14 weeks, respectively, after the onset of CRAO. In 4/7 cases, resolution occurred within 2 days after the removal of the external factor. Surprisingly, Wilson et al.^25^ reported that after 4 days, the hallucinations resolved spontaneously despite ongoing monocular occlusion. Considering the duration of hallucinations, calculated as the time from onset to cessation, the average time span among the seven cases where it could be determined was 17.6 days (range 2 h–96 days).

The frequency of hallucinations, reported in only four articles, was several times daily,^19^, ^20^, ^22^, ^23^ with individual episodes lasting seconds^19^, ^22^ to 1–2 min,^23^ except for Beaulieu et al.^20^ who reported individual episodes lasting 2–5 min. The outcome of the hallucinations, despite being a fundamental aspect of the assessment, was reported in only three cases, of which two were moderately distressing^24^, ^25^ and one was neutral to the patient.^22^

**FIGURE 4:**
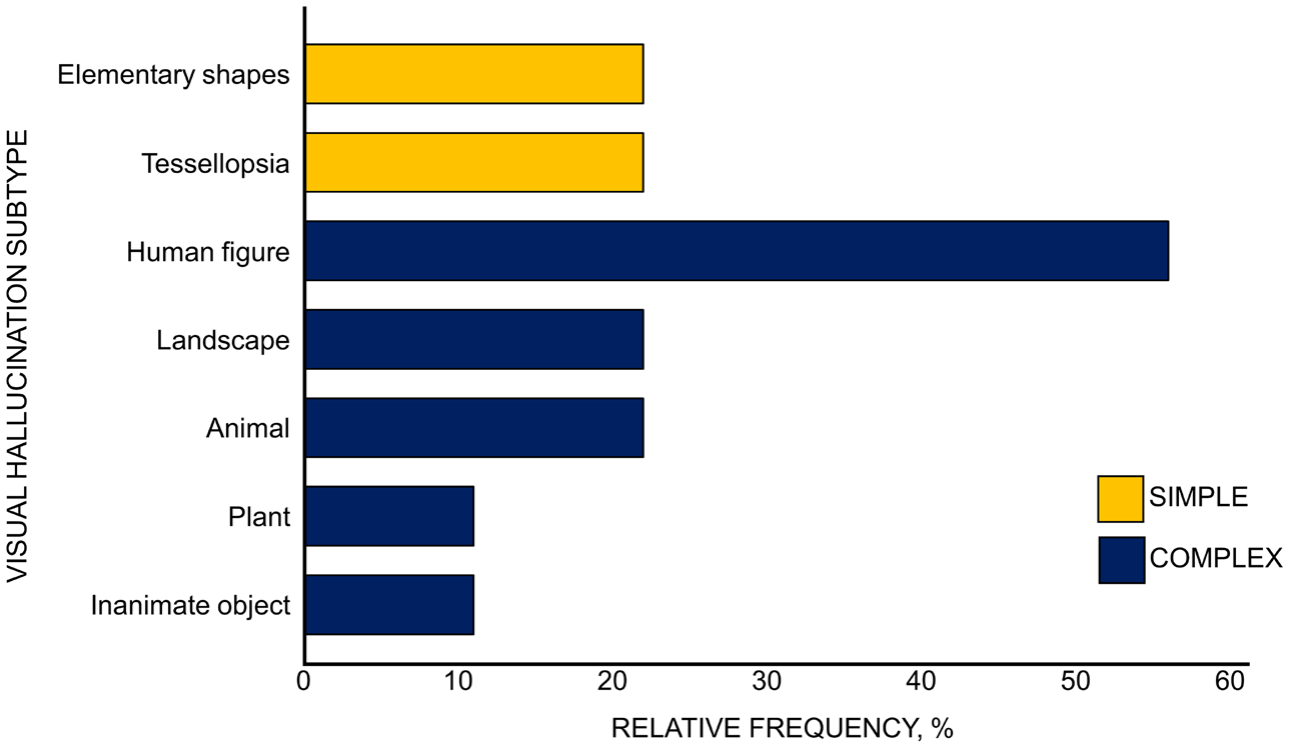
Relative frequencies (%) of visual hallucination subtypes in nine cases of Charles Bonnet Syndrome associated with unilateral visual loss included in this review.

The reported hallucinations were categorised as either simple or complex, both further divided into several subcategories. The relative frequency of each hallucination subtype is depicted in Figure 4. Simple hallucinations of elementary shapes and tessellopsia were observed in 22% of cases.^20^, ^23^ Complex hallucinations were present in 100% of patients, with human figures being the most common (56%): ‘an unfamiliar person walk’,^19^ ‘shaped figures creeping across the room’,^20^ ‘small, faceless children playing in the snow’,^25^ ‘people walking towards a pyramid carrying a person’,^26^ ‘four figures around her’.^21^ Additionally, landscape^22^, ^26^ and plant^20^, ^23^ hallucinations occurred in 22% of cases, while animal^21^ and inanimate objects^20^ appeared in 11%. The hallucinations were described as formed, complex, repetitive and stereotyped, with insight preserved and no primary or secondary delusions.

## DISCUSSION

Current literature on CBS states that ‘significant vision loss’ is necessary before the onset of hallucinations for a diagnosis of CBS, suggesting that a loss of over 60% of vision is required to develop the condition.^12^ However, this statement might be arbitrary given the lack of systematic, large‐scale investigations exploring the degree of visual impairment necessary to experience CBS.

A case characterised by typical CBS hallucinations is presented here. The visual hallucinations were complex, consisting of formed images and abstract patterns in motion, adhering to the criteria of Gold and Rabin.^27^ The visual hallucinations appeared immediately after the onset of visual impairment and remained extremely frequent even 2 years after the acute event. Since vision loss was complete in the right eye while the left eye maintained excellent visual function, this does not meet the ‘significant vision loss’ criterion. Indeed, under UK legislation, the loss of vision in one eye does not allow certification as sight impaired or severely sight impaired if the other eye maintains excellent functionality, and is not legally considered to represent a sight impairment.^28^ Additionally, it would be extremely difficult to quantify the percentage of vision loss at which CBS may occur, as this would require weighting and defining multiple aspects of vision. For instance, in the presented case, the patient experienced not only a loss of visual acuity (VA) but also a loss of binocular three‐dimensional vision. This makes it challenging to assess the relative significance of the loss of three‐dimensional vision compared to the loss of VA in percentage terms.

Unlike the cases included in the review that had follow‐up (7/9 cases), all of which showed resolution of visual hallucinations, a follow‐up evaluation was not performed here. However, at the time of the evaluation, the patient had already experienced persistent visual hallucinations for 2 years without attenuation since their onset. Had the other cases been followed up for longer, they may have observed a return of the CBS symptoms.

The ‘deafferentation theory’ posits that reduced visual input to the brain leads to decreased cortical inhibition, resulting in spontaneous hyperactivity within visual cortical areas.^8^, ^29^, ^30^ The precise mechanisms through which deafferentation triggers the onset of hallucinations remain unclear.^31^ The present findings demonstrate that vision loss in one eye only can disrupt sensory input to the visual cortex sufficiently to precipitate hallucinations. A detailed analysis of how cases of CBS associated with unilateral vision loss aligns with potential underlying mechanisms is beyond the scope of this study.

Given the unclear pathogenesis, describing a specific phenomenology is essential for correctly defining CBS.^27^, ^32^ From a phenomenological perspective, in the presented case and the other nine cases reviewed, visual hallucinations that developed after vision loss in one eye—while the fellow eye maintained excellent visual function—are identical to those seen in CBS associated with bilateral vision loss. The phenomenological framework is consistent with that described by ffytche et al.,^9^ who outlined it as a phenomenological syndrome with macular disease as a prototypical disorder. Therefore, a loss of more than 60% of vision seems unlikely to be a necessary criterion for experiencing CBS and may contribute to the underdiagnosis. Additionally, the present case and the nine cases in the literature fit perfectly the current ICD‐11 definition of CBS as the experience of complex visual hallucinations in a person who has experienced ‘partial or complete loss of vision’. In fact, all 10 can be defined as ‘partial’ visual loss.^6^

An interesting aspect to analyse is that in 5/9 reviewed cases, external factors such as eye patching,^20^, ^23^ Frost suture^20^, ^24^ and reconstruction with a Hughes procedure^25^ following surgery contributed to the development of hallucinations. Campbell et al.^1^ proposed that CBS results from the interplay of multiple factors. More specifically, the ‘phantom vision’ that develops as a result of CBS is the expression of abnormal activity in the visual brain due to reduced visual input and the ‘vulnerability’ of the visual brain, which can be considered mainly determined by vascular damage.^1^, ^11^ Additionally, visual input is not only influenced by visual impairment but also by visual sensory deprivation (e.g., external factors like eye patching). These factors compound the effects of visual impairment by reducing cortical input further. For example, Beaulieu et al.^20^ reported a case of CBS associated with eye patching and a Frost suture following eyelid surgery. However, the patient also had exposure keratopathy, which affected light transmission and thereby contributed to the visual sensory deprivation. Hence, it is important not to consider visual function solely in terms of VA, which assesses only the functionality of the central retina (≈0.5 mm or 2.75°).^33^ When evaluating visual loss, multiple aspects of vision should be taken into account.

Interestingly, Merabet et al.^34^ showed that prolonged complete bilateral visual deprivation could elicit release‐like visual experiences in non‐visually impaired subjects. Meanwhile, Wackermann et al.^35^ demonstrated that in people with preserved vision, exposure to a bilateral, uniform and unstructured visual field (Ganzfeld) can trigger complex visual hallucinations through perceptual deprivation. However, it has not yet been explored whether monocular deprivation could trigger either visual hallucinations with release features (when using a unilateral blindfold) or Ganzfeld‐induced phenomena (when exposed to a Ganzfeld) in subjects without visual impairment. Such research could contribute to a better understanding of the neural mechanisms underlying unilateral CBS.

A potential limitation of this study may be that the inclusion criteria defined two levels of VA depending on whether it was best corrected or if it was non‐specified (Table 1). However, in all cases, no ophthalmic pathology was reported in the fellow eye. Therefore, the assumption was that in cases where it was not specified, the slight VA deficit was caused by an uncorrected refractive error. In two cases, the VA was explicitly reported as uncorrected. Gander et al.^24^ indicated a VA of +0.30 logMAR, but since it was uncorrected, the report was still included in the review. Similarly, Khadavi et al.^23^ reported an uncorrected VA of 20/20 (logMAR = 0.00), which was also included.

To reduce underdiagnosis, we propose that CBS should be screened in anyone who has experienced any form or degree of vision loss. Clinicians should thoroughly investigate the presence of visual hallucinations through targeted questioning and, if possible, utilise questionnaires such as the UM‐PDHQ,^15^ the North‐East Visual Hallucinations Interview (NEVHI)^36^ and the Charles Bonnet Syndrome Screening Questionnaire (QR‐SCB).^37^ Proper screening is crucial because, as outlined in the latest guidelines for the management of visual hallucinations in ophthalmological disease, pre‐emptive questioning and forewarning might be effective in reducing emotional impact or distress.^38^

In conclusion, it is important to recognise that CBS may not be associated with severe bilateral visual impairment, as complex visual hallucinations are a common outcome when visual cortical areas are deprived of input.^1^ Therefore, it is essential to make the diagnosis more inclusive. We speculate that there is a group of patients with CBS who are not diagnosed due to the misconception that higher degrees of visual impairment are a prerequisite, as suggested by the current literature.^12^ Introducing this new diagnostic perspective could decrease underdiagnosis and result in a higher number of reported cases. This represents a critical advancement in furthering the understanding of the syndrome and should encourage greater screening of CBS in all ophthalmic and other related clinics.

## AUTHOR CONTRIBUTIONS

**Giovanni Forte:** Conceptualization (lead); formal analysis (lead); writing – original draft (lead). **Natalie Assaf:** Formal analysis (equal); investigation (equal); writing – review and editing (equal). **Paolo Forte:** Formal analysis (equal); writing – review and editing (equal). **Jasleen K. Jolly:** Conceptualization (lead); formal analysis (lead); funding acquisition (lead); investigation (lead); methodology (lead); supervision (lead); writing – review and editing (lead).

## FUNDING INFORMATION

This study was funded by an ARU Science and Engineering QR grant. GF was funded by Ghislieri College (St John's College Undergraduate Research Fellowship, Cambridge, UK).

## CONFLICT OF INTEREST STATEMENT

The authors report no conflicting interests.
